# Hepatic Ectopic Pregnancy: A Diagnostic Challenge Highlighted by Multimodal Imaging

**DOI:** 10.3390/jcm15062388

**Published:** 2026-03-20

**Authors:** Puja Punukollu, Lindsey Grater, Claudia Szlek, Rebecca Joseph, John Lue, James Maher, Lawrence Devoe

**Affiliations:** Department of Obstetrics and Gynecology, Medical College of Georgia, Augusta, GA 30912, USA

**Keywords:** hepatic ectopic pregnancy, ectopic pregnancy, multimodal imaging, methotrexate

## Abstract

Background: Ectopic pregnancy occurs in about 1–2% of all pregnancies, with 95% implanting in the fallopian tubes. Hepatic implantation is one of the rarest and most dangerous forms of abdominal ectopic pregnancy. Its diagnosis is often delayed because of nonspecific symptoms, and it is also often difficult for routine ultrasound imaging to visualize ectopic pregnancy sites that are not in the pelvis. Since this type of pregnancy carries a risk of severe hemorrhage, early identification is crucial. Case: A 30-year-old woman, gravida 3 para 2, presented with a serum β-hCG of 66,408 mIU/mL, but no intrauterine pregnancy was detected on ultrasound imaging. At an outside facility, a laparoscopy was performed, which also failed to show a pelvic ectopic pregnancy. The patient then received her first dose of methotrexate and was subsequently transferred to a tertiary care center for further evaluation. MRI and liver ultrasound showed a 2.3 cm subcapsular lesion in segment 5 of the liver that was suspicious for a hepatic ectopic pregnancy. However, these imaging studies could not exclude a gestational trophoblastic disease or hepatic neoplasm. A dilation and curettage revealed no trophoblastic tissue. The patient next received two additional doses of methotrexate on hospital days 4 and 7 due to an inadequate decline in interval β-hCG; β-hCG levels declined gradually but steadily over several months until they became undetectable and indicated a successful medical treatment of her hepatic ectopic pregnancy. Conclusions: This case highlights the complex diagnostic and treatment challenges presented by a hepatic ectopic pregnancy. Multimodal imaging, serial monitoring of β-hCG levels, and the engagement of a multidisciplinary team were essential factors in achieving a safe, nonsurgical, and successful resolution of this condition. When a pregnancy of unknown location is suspected, extended imaging studies are critical tools for patient evaluation after initial imaging studies and laparoscopy are inconclusive.

## 1. Introduction

Ectopic implantation of a fertilized ovum outside the uterine cavity occurs in approximately 1–2% of all pregnancies and remains a major cause of maternal morbidity and mortality worldwide [1]. The fallopian tube is the most common site of implantation [2], but rare cases of abdominal ectopic pregnancies have been documented, including implantations on the bowel, omentum, spleen, and liver. Abdominal ectopic pregnancies carry a significantly higher risk of morbidity and mortality compared to tubal ectopic pregnancies, with maternal mortality rates up to 7.7 times greater and as much as 90 times higher than intrauterine pregnancies [3]. Hepatic ectopic pregnancy is one of the rarest forms of abdominal pregnancy and presents unique diagnostic and therapeutic challenges because of its unusual location and the high vascularity of the liver.

Hepatic ectopic pregnancy is difficult to diagnose because its clinical symptoms are often nonspecific, and pelvic imaging can often be inconclusive. Persistently high serum beta-human chorionic gonadotropin (β-hCG) levels without visualization of either an intrauterine pregnancy or a pelvic mass should raise the suspicion of rare abdominal implantation sites. Advanced imaging techniques, such as magnetic resonance imaging (MRI) and targeted ultrasound of the liver, are essential for accurate localization of such pregnancies. Treatment options vary and frequently involve a multidisciplinary team, including obstetrics, gynecologic oncology, and interventional radiology. This case report describes the diagnostic methods, clinical management, and follow-up of a suspected hepatic ectopic pregnancy successfully treated with systemic methotrexate. It also emphasizes the importance of utilizing carefully targeted imaging modalities and collaborative care to enhance outcomes in these rare but potentially life-threatening cases.

## 2. Case Presentation

A 30-year-old woman, gravida 3, para 2, was initially evaluated at a rural hospital for early pregnancy assessment at 8 weeks and 2 days by last menstrual period. She had minor LLQ abdominal bleeding throughout the pregnancy, but denied any vaginal bleeding, nausea, vomiting, or syncope. Her initial serum β-hCG level was 66,408 mIU/mL. Initial laboratory evaluation was largely unremarkable, with a hemoglobin of 12.3 g/dL, white blood cell count of 10.4 × 10^3^/µL with a normal differential, and creatinine of 0.61 mg/dL. Liver-associated enzymes were within or near reference range (AST 44 U/L, ALT 27 U/L, alkaline phosphatase 51 U/L). Electrolytes were notable for mild hypokalemia (potassium 3.1 mmol/L), with sodium at 141 mmol/L and chloride at 105 mmol/L; blood urea nitrogen was 8 mg/dL. Red blood cell count was 4.34 ×10^6^/µL. A transvaginal ultrasound (TVUS) was performed using a standard high-frequencies endovaginal probe, revealing the absence of an intrauterine pregnancy (IUP), and an endometrial thickness of 23 mm, a normal left ovary, and a right ovary with a complex cystic area measuring approximately 2.0 cm adjacent to it. There were also complex areas posterior and medial to the right ovary measuring 4.0 × 1.5 × 2.9 cm and 1.8 × 1.3 cm, respectively, consistent with benign ovarian cysts.

**Figure 1:**
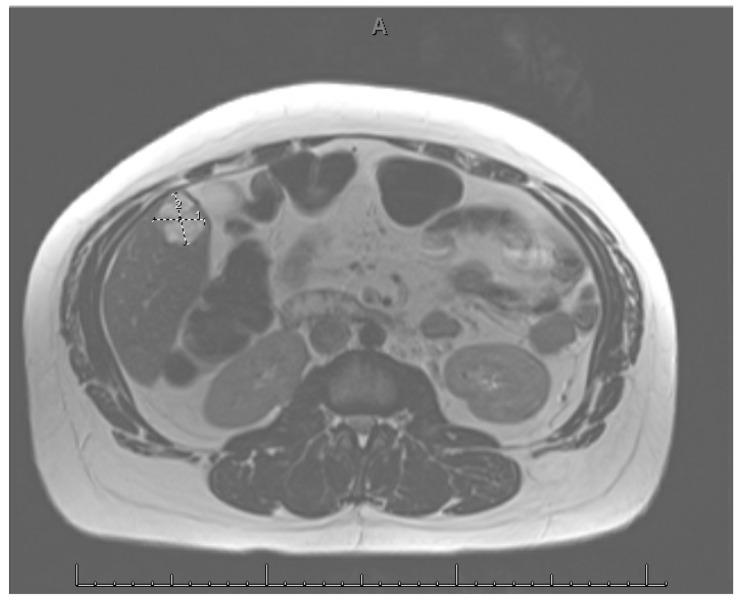
Abdominal MRI. Right Hepatic Lobe Mass.

A diagnostic laparoscopy was performed and showed no ectopic pregnancy or mass within the pelvis. Endometriosis and adhesions of the left ovary within the ovarian fossa were present. Initially a single dose of methotrexate (50 mg/m^2^) was administered with plans for repeat dosing on days 4 and 7. Magnetic resonance imaging (MRI) was performed, which identified an indeterminate, heterogeneous mass in the inferior right hepatic lobe measuring 2.7 × 2.5 cm that raised suspicion for a hepatic ectopic pregnancy (Figure 1). A hepatic ultrasound confirmed a subcapsular mass in the inferior right hepatic lobe measuring 2.3 × 2.1 × 1.9 cm. Due to the potential catastrophic risk of rupture of this mass, the patient was transferred to a tertiary care center for further evaluation and treatment. Upon transfer, the patient indicated some right upper quadrant pain without acute changes and intermittent nausea.

**Figure 2:**
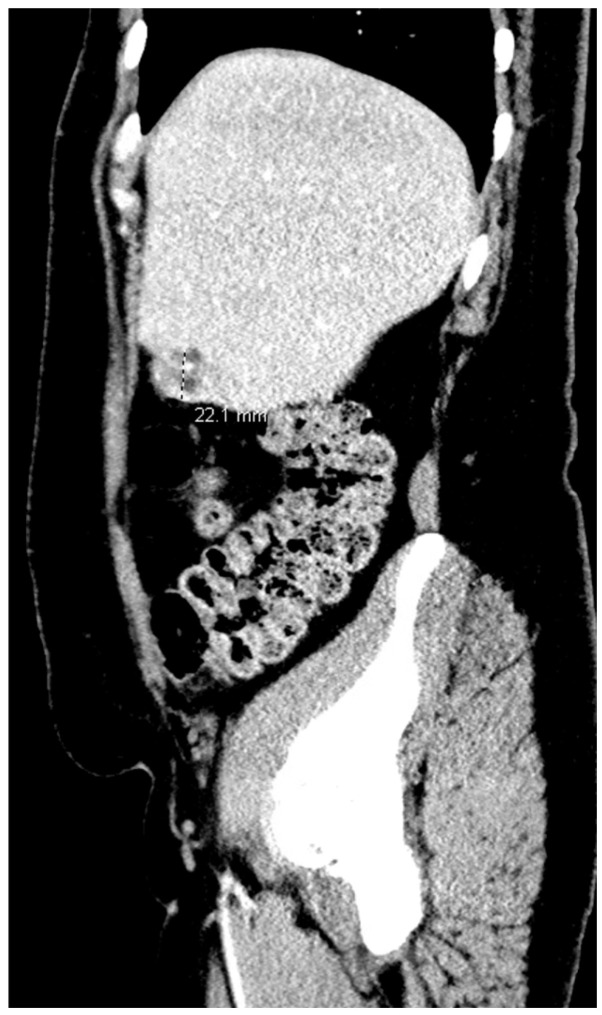
CT Hepatic Segment 5.

Repeated imaging at the tertiary hospital, using computed tomography (CT) of the abdomen and pelvis, showed a heterogeneously enhancing, mainly fluid-filled subcapsular lesion in hepatic segment 5, measuring 2.5 × 2.3 cm, but no additional lesions or signs of rupture were detected (Figure 2). Repeat β-hCG levels remained persistently elevated with minimal overall change: 66,408 mIU/mL initially, 61,966 mIU/mL early on day 2, 65,396 mIU/mL on day 3, 64,116 mIU/mL on day 4, and 63,156 mIU/mL on day 7. Methotrexate was administered as a multidose regimen on day 1, day 4, and day 7 in response to persistently elevated β-hCG levels; leucovorin rescue was not administered.

The gynecologic oncology service was consulted and formulated a list of differential diagnoses that included hepatic ectopic pregnancy, metastatic gestational trophoblastic disease from a primary gynecologic source, or a primary liver tumor that was producing beta-HCG.

On the day following the last methotrexate dose, the patient underwent dilation and curettage (D&C) in an attempt to rule out a uterine origin for β-hCG–secreting tissue. Given the lack of appropriate biochemical response to methotrexate prior to D&C, the patient was counseled that if D&C pathology supported an intrahepatic ectopic pregnancy, gynecologic oncology would recommend medical management with actinomycin D due to failure of methotrexate therapy. She was also counseled that if pathology suggested choriocarcinoma/GTN, aggressive chemotherapy would be required, including a combination of inpatient and outpatient management, given a high-risk WHO score if the hepatic mass represented metastasis.

**Figure 3:**
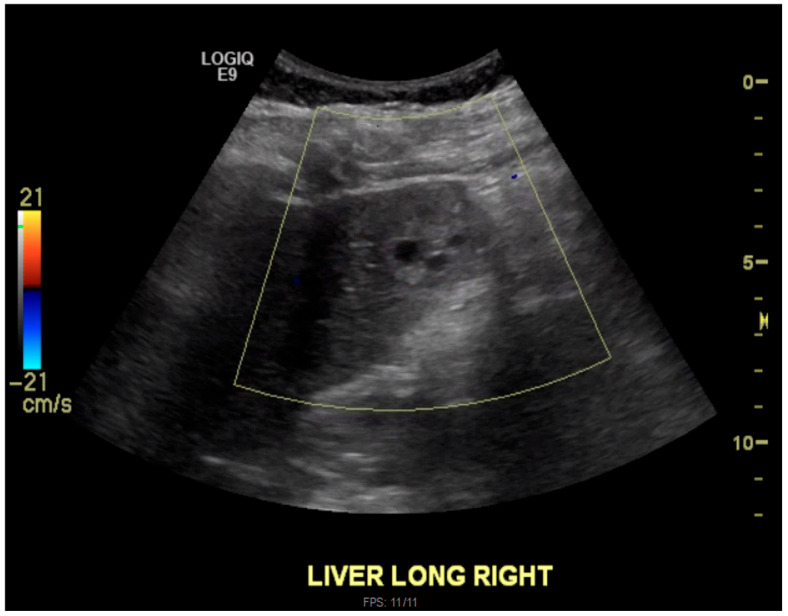
Liver ultrasound. Longitudinal section. Right lobe.

The pathology report revealed decidualized endometrium, with no evidence of chorionic villi or fetal tissue. A follow-up ultrasound of the right upper quadrant on day 10 revealed that the heterogeneously hyperechoic lesion with internal cystic areas within segment 5 of the liver was still present and was consistent with the previous CT and MRI findings (Figure 3). At that time, imaging could not definitively differentiate between a hepatic ectopic pregnancy and gestational trophoblastic neoplasia (GTN) metastasis.

Gynecologic Oncology deemed her medically stable for outpatient follow-up due to declining β-hCG and stable patient presentation. The patient chose to go home and follow up with her primary physician. A Nexplanon contraceptive implant was placed at the time of discharge to prevent pregnancy during ongoing β-hCG monitoring and hepatic lesion surveillance. The patient was specifically counseled regarding reliable contraception both to avoid pregnancy soon after methotrexate exposure due to neural tube defect risk and to allow accurate interpretation of β-hCG trends, as GTN was considered unlikely but not entirely excluded.

Following the initial inpatient course, β-hCG was monitored closely with serial outpatient testing. After the initial post–D&C decline to 49,557 mIU/mL, levels continued to decrease to 38,847 mIU/mL and 38,200 mIU/mL over the subsequent days. Continued outpatient surveillance demonstrated a progressive decline to 11,908 mIU/mL by approximately three weeks post-treatment, 1556 mIU/mL by five weeks, and 536.3 mIU/mL by six weeks; β-hCG further decreased to 115 mIU/mL by approximately eight weeks and reached a nadir in the 50–60 mIU/mL range over the following several weeks, with minor low-level fluctuations. Ultimately, β-hCG declined to near-undetectable levels (<1 mIU/mL) by approximately five months post-treatment and remained suppressed on subsequent follow-up testing, indicating sustained biochemical resolution.

Interval imaging during follow-up demonstrated concordant anatomic improvement. Approximately two months after initial presentation, follow-up abdominal ultrasound demonstrated a normal-appearing liver without a discrete residual hepatic lesion. Several months later, repeat cross-sectional imaging with computed tomography (CT) of the chest, abdomen, and pelvis demonstrated a marked interval decrease in the size and enhancement of the previously identified hepatic lesion, measuring approximately 1.2 × 1.6 cm compared to its original dimensions, with minimal residual intralesional enhancement, consistent with treatment response. Additional surveillance imaging, including pelvic ultrasound and brain MRI obtained during follow-up, revealed no findings that indicated concern for metastatic disease. Serial imaging over subsequent months confirmed continued regression without recurrence, supporting resolution of a gestational focus rather than an underlying hepatic neoplasm. Liver function tests remained largely stable throughout follow-up, with no clinically significant or progressive abnormalities observed.

Given D&C pathology without chorionic villi, the continued decline in β-hCG without additional chemotherapy, and interval radiographic resolution of the hepatic lesion, GTN metastasis was ultimately considered unlikely and was systemically ruled out. The management plan included close outpatient surveillance without further chemotherapy with ongoing follow-up with gynecologic oncology through 80 telehealth contacts over 6 months, during which serial β-hCG levels and hepatic ultrasounds were monitored to ensure resolution of the hepatic lesion and normalization of hormone levels. No treatment-related adverse events, hemorrhagic complications, or need for additional intervention occurred during follow-up; however, intermittent delays in laboratory surveillance were noted, necessitating repeated outreach and reinforcing the importance of continued β-hCG monitoring

## 3. Discussion

Hepatic ectopic pregnancy is a very rare form of ectopic gestation that presents significant diagnostic and therapeutic challenges. A recent systematic review identified fewer than 50 reported cases worldwide, the majority described as isolated case reports spanning several decades, reflecting both the rarity of the condition and the absence of standardized diagnostic or management guidelines [4]. In reported cases, clinical presentation is often nonspecific and may include right upper quadrant or epigastric pain, vaginal bleeding, nausea, or signs of hemoperitoneum, although some cases are discovered incidentally during evaluation of pregnancy of unknown location. In this case, elevated β-hCG levels and the absence of intrauterine pregnancy led to the diagnosis and treatment plan for ectopic pregnancy that became suspicious for an abdominal ectopic pregnancy when neither ultrasound imaging nor laparoscopy showed a pelvic ectopic pregnancy. Multiple imaging modalities, including ultrasound, MRI, and CT scans, were crucial in finally identifying the hepatic lesion.

There is a strong similarity between the behavior of early trophoblasts and that of invading cancer [5]. Once pregnancies are expelled into the peritoneal cavity, they may implant on neighboring tissues or enter the general flow of fluid within the peritoneal cavity, eventually implanting in the right infrahepatic region, where invading trophoblastic tissue establishes a circulation with the maternal vascular tree. Disruption of this circulation can lead to severe hemorrhage.

Hepatic ectopic pregnancy can present on ultrasound with various manifestations, including: Pregnancy Sac Type, which is characterized by the presence of a distinct gestational sac, potentially containing a visible fetal pole and exhibiting fetal cardiac activity; Thick-walled cystic nodular type, appearing as a cystic mass within the liver with a thickened wall; Heterogeneous hyperechoic type, presenting as a mass with mixed echogenicity; and Cystic-solid mixed echo type, combining features of both cystic and solid structures, with fluid-filled and solid components visible on ultrasound [6,7]. In this case, failure to demonstrate a distinct gestational sac, fetal pole, and cardiac activity in conjunction with markedly elevated β-hCG levels also raised the possibility of either GTN or a primary hepatic neoplasm. Because definitive sonographic pregnancy structures are frequently absent, the diagnosis in many reported cases is supported by a composite of imaging findings, serial β-hCG behavior, and clinical course rather than histologic confirmation.

Primary surgical management is complicated by the vascularity of the liver and the risk of life-threatening hemorrhage that can be provoked by using this strategy. Methotrexate was used as initial medical therapy for the presumed ectopic pregnancy, but when the patient’s β-hCG serum levels failed to decline after receiving three doses of methotrexate, other diagnoses were considered, including gestational trophoblastic neoplasia and hepatic tumors. Multidisciplinary care and serial β-hCG monitoring guided treatment decisions, ultimately allowing for successful medical management. Historically, most reported hepatic ectopic pregnancies have been managed with open surgical removal, often via exploratory laparotomy, although more recent cases increasingly describe laparoscopic approaches when feasible. Conservative approaches using methotrexate remain uncommon overall, but contemporary reports support its growing role either as primary therapy in carefully selected stable patients or as a bridging strategy to reduce bleeding risk prior to planned resection [4].

Systemic methotrexate was selected as the initial treatment strategy due to the patient’s hemodynamic stability, relatively small size of the hepatic lesion (<3 cm), absence of rupture, and the high risk of life-threatening hemorrhage associated with surgical resection of hepatic ectopic pregnancies. A commonly used single-dose methotrexate regimen has an overall reported success rate of approximately 88% (95% CI 86–90%), with treatment success typically defined as a ≥15% decline in serum β-hCG over the standard follow-up interval [8]. The subcapsular location of the lesion further increased the potential bleeding risk with operative intervention.

A key limitation of this case is that histologic confirmation of trophoblastic tissue within the hepatic lesion was not obtained; therefore, the diagnosis remains presumptive based on imaging, exclusion of alternative etiologies, and the subsequent biochemical and radiographic response. Metastatic gestational trophoblastic neoplasia was systematically considered given markedly elevated β-hCG and an apparent hepatic mass; however, endometrial sampling did not demonstrate chorionic villi or fetal tissue, and β-hCG declined steadily without multi-agent chemotherapy, making GTN less likely. A primary hepatic tumor producing β-hCG was also considered, but interval resolution of the lesion on serial imaging alongside normalization of β-hCG argued against an underlying neoplasm. Explicitly framing the diagnosis as presumptive while detailing this exclusion process is important because management pathways diverge substantially across these differentials.

Because markedly elevated β-hCG with a hepatic lesion raises concern for gestational trophoblastic neoplasia with hepatic involvement, we approached the differential systematically. Standard GTN evaluation emphasizes correlation of serial β-hCG trends with uterine assessment and staging studies and includes assessment of end-organ function (renal, hepatic, marrow) and chest imaging given the propensity for pulmonary metastases. In our case, endometrial sampling demonstrated decidualized endometrium without chorionic villi or fetal tissue, which argued against an intrauterine gestational source. In addition, β-hCG declined steadily without multi-agent chemotherapy, and the hepatic lesion regressed on serial imaging, making metastatic GTN less likely [9]. A primary hepatic tumor producing β-hCG was also considered; however, the concordant biochemical resolution and radiographic regression favored a gestational focus rather than neoplasia.

This case highlights the importance of thorough imaging and biochemical monitoring in managing unusual ectopic pregnancies, as well as the successful collaboration in care between the rural primary care team and the tertiary referral team, facilitated through close contact by using telehealth modalities. This case supports that selected, hemodynamically stable patients with small, unruptured hepatic lesions may be managed conservatively with close surveillance, while maintaining readiness to escalate to interventional radiology or surgery if hemorrhage or nonresponse occurs.

## 4. Conclusions

Hepatic ectopic pregnancy represents a rare and life-threatening condition that poses significant diagnostic and therapeutic challenges. Early recognition through multimodal imaging and serial β-hCG measurement is vital for timely diagnosis. This case illustrates that a multidisciplinary, nonsurgical management strategy, incorporating methotrexate and close monitoring, can be effective in selected patients, thereby minimizing the morbidity associated with hepatic surgery. Continued awareness and reporting of such cases are essential to improve the understanding of and to optimize outcomes in this rare clinical scenario.

## Data Availability

Data are contained within the article.
